# Supplementary material for: Dietary similarity among jaguars (Panthera onca) in a high-density population
Source: PLoS One. 2022 Oct 10;17(10):e0274891. doi: 10.1371/journal.pone.0274891 (PMC9550027; doi:10.1371/journal.pone.0274891)
Supplement: S1 Table — Scat-based studies of jaguar diet from published and unpublished sources, showing the study country, the number of years during which scat collection was conducted, the method for assigning felid species to the scats (‘signs’ includes tracks, scrapes, and resting places), whether the scats were genotyped to the level of individual jaguar, whether criteria were applied to exclude potentially non-independent scats (those presumed to have been produced by the same individual feeding from the same carcass), and the number of scats that were ultimately analysed to describe the diet. (DOCX) [file pone.0274891.s001.docx]

**Dietary similarity among jaguars (*Panthera onca*) in a high-density population**

Rebecca J. Foster^1*^ and Bart J. Harmsen^1^

^1^ Panthera, New York, New York, USA

^*^ Corresponding author

Email: [rfoster@panthera.org](about:blank)

**Supporting Information**

**S1 Table. Species identification and sample size in scat-based studies of jaguar diet.**

Scat-based studies of jaguar diet from published and unpublished sources, showing the study country, the number of years during which scat collection was conducted, the method for assigning felid species to the scats (‘signs’ includes tracks, scrapes, and resting places), whether the scats were genotyped to the level of individual jaguar, whether criteria were applied to exclude potentially non-independent scats (those presumed to have been produced by the same individual feeding from the same carcass), and the number of scats that were ultimately analysed to describe the diet.

| **Country** | **Survey years** | **Felid species identification** | **Individual genotyping** | **Exclude non-independent scats** | **Number of scats analysed for diet** | **Ref** |
| --- | --- | --- | --- | --- | --- | --- |
| Argentina | not stated | not stated | no | no | 246 | [1] |
| Belize | 4 | genetic | no | no | 79 | [2] |
| Belize | 4 | genetic | no | no | 322 | [3] |
| Belize | 1 | genetic | yes | no | 9 | [4] |
| Belize | not stated | not stated | no | no | 228 | [5] |
| Belize | 1 | hair, genetic | no | no | 23 | [6] |
| Brazil | 5 | signs, hair | no | no | 73 | [7] |
| Brazil | 4 | signs, scat size/morphology | no | no | 35 | [8] |
| Brazil | 5 | signs, hair | no | no | 51 | [9] |
| Brazil | 2 | signs | no | no | 149 | [10] |
| Brazil | 2 | signs, hair | no | no | 134 | [11] |
| Brazil | 6 | genetic | no | no | 32 | [12] |
| Brazil | 2 | signs, scat size/morphology, hair | no | no | 13 | [13] |
| Brazil | 1 | signs, hair | no | yes | 101 | [14] |
| Brazil | 3 | signs, hair | no | no | 32 | [15] |
| Brazil | 1 | genetic | yes | no | 50 | [16] |
| Brazil | 4 | signs, collar data | no | yes | 125 | [17] |
| Brazil | 2 | signs, scat size/morphology | no | no | 29 | [18] |
| Brazil | not stated | signs, scat size/morphology | no | no | 18 | [19] |
| Brazil | 6 | genetic | yes | no | 35 | [20] |
| Brazil | 2 | hair, genetic | no | no | 16 | [21] |
| Brazil | 2 | signs, scat size/morphology | no | no | 1 | [22] |
| Costa Rica | 5 | signs, scat size/morphology | no | no | 75 | [23] |
| Costa Rica | 3 | signs | no | no | 18 | [24] |
| Costa Rica | 2 | signs, scat size/morphology | no | no | 22 | [25] |
| Costa Rica | 1 | not stated | no | no | 15 | [26] |
| Costa Rica | 1 | not stated | no | no | 5 | [27] |
| Guatemala | 12 | signs | no | no | 93 | [28] |
| Guatemala | 2 | genetic | no | no | 76 | [29] |
| Mexico | 5 | signs | no | no | 37 | [30] |
| Mexico | 2 | genetic | no | no | 23 | [31] |
| Mexico | 2 | genetic | no | no | 5 | [32] |
| Mexico | 10 | signs, scat size/morphology | no | no | 45 | [33] |
| Mexico | 8 | signs, scat size/morphology, camera trap data, bile acid | no | no | 13 | [34] |
| Mexico | 4 | signs, scat size/morphology, bile acid | no | no | 54 | [35] |
| Mexico | 3 | genetic | no | no | 43 | [36] |
| Mexico | 4 | signs | no | no | 50 | [37] |
| Mexico | 6 | signs, scat size/morphology | no | no | 27 | [38] |
| Mexico | 2 | genetic | no | no | 11 | [39] |
| Paraguay | 1 | signs, scat size/morphology, camera trap data, collar data | no | no | 41 | [40] |
| Paraguay | 3 | bile acid | no | no | 106 | [41] |
| Peru | 4 | signs, hair, camera trap data, collar data | no | yes | 25 | [42] |
| Peru | 2 | signs | no | no | 13 | [43] |
| Venezuela | 1 | genetic | no | no | 3 | [44] |
| Venezuela | 3 | signs | no | no | 42 | [45] |

**References**

1. Perovic PG. Conservación del jaguar en el noroeste de Argentina. In: Medellín RA, Equihua C, Chetkiewicz CLB, Crawshaw PGJ, Rabinowitz A, Redford KH, et al., editors. El jaguar en el nuevo milenio. Mexico: Fondo de Cultura Económica; 2002. p. 465-76.

2. Figueroa OA. The ecology and conservation of jaguars (Panthera onca) in central Belize: Conservation status, diet, movement patterns and habitat use. Florida, USA: University of Florida; 2013.

3. Foster RJ, Harmsen BJ, Valdes B, Pomilla C, Doncaster CP. Food habits of sympatric jaguars and pumas across a gradient of human disturbance. Journal of Zoology. 2010;280(3):309-18. doi: 10.1111/j.1469-7998.2009.00663.x. PubMed PMID: WOS:000274412100010.

4. Mesa-Cruz JB, Brown JL, Waits LP, Kelly MJ. Non-invasive genetic sampling reveals diet shifts, but little difference in endoparasite richness and faecal glucocorticoids, in Belizean felids inside and outside protected areas. J Trop Ecol. 2016;32:226-39. doi: 10.1017/s0266467416000213. PubMed PMID: WOS:000377296800005.

5. Rabinowitz AR, Nottinghamn BG. Ecology and behaviour of the jaguar (Panthera onca) in Belize, Central America. Journal of Zoology. 1986;210:149-59.

6. Weckel M, Giuliano W, Silver S. Cockscomb revisited: Jaguar diet in the Cockscomb Basin Wildlife Sanctuary, Belize. Biotropica. 2006;38(5):687-90. doi: 10.1111/j.1744-7429.2006.00190.x. PubMed PMID: WOS:000239634900015.

7. Crawshaw PG, Mähler JK, Indrusiak C, Cavalcanti SMC, Pitman RL, Silvius KM. Ecology and conservation of the jaguar (Panthera onca) in Iguaçu National Park, Brazil. In: Silvius KM, Bodmer RE, Fragoso JMV, editors. People in nature: wildilfe conservation in South and Central America. New York: Columbia University Press; 2004. p. 271-85.

8. Dalponte JC. Dieta del jaguar y depredación de ganado en el norte del Pantanal, Brasil. In: Medellín RA, Equihua C, Chetkiewicz CLB, Crawshaw PGJ, Rabinowitz A, Redford KH, et al., editors. El jaguar en el nuevo milenio. Mexico: Fondo de Cultura Económica; 2002. p. 209-22.

9. de Azevedo FCC. Food habits and livestock depredation of sympatric jaguars and pumas in the Iguacu National Park area, south Brazil. Biotropica. 2008;40(4):494-500. doi: 10.1111/j.1744-7429.2008.00404.x. PubMed PMID: WOS:000257717500014.

10. de Azevedo FCC, Murray DL. Spatial organization and food habits of jaguars (Panthera onca) in a floodplain forest. Biological Conservation. 2007;137(3):391-402. doi: 10.1016/j.biocon.2007.02.022. PubMed PMID: WOS:000247775600008.

11. de Oliveria GE. Ecologia alimentar da onça-pintada (Panthera onça) na subregião do Pantanal de Miranda [MSc]. Brazil: Universidade Federal de Mato Grosso Do Sul; 2009.

12. do Prado DM. Dieta e relação de abundância de Panthera onca e Puma concolor com

suas espécies-presa na Amazônia central. Brazil: Instituto Nacional de Pesquisas da

Amazônia, Brazil; 2010.

13. Facure K, Giaretta AA. Food habits of carnivores in a coastal Atlantic Forest of southeastern Brazil. Mammalia. 1996;60(3):499-502.

14. Garla RC, Setz EZF, Gobbi N. Jaguar (Panthera onca) food habits in Atlantic rain forest of southeastern Brazil. Biotropica. 2001;33(4):691-6. doi: 10.1111/j.1744-7429.2001.tb00226.x. PubMed PMID: WOS:000173456300014.

15. Leite MRP, Galvão F. El jaguar, el puma y el hombre en tres áreas protegidas del bosque atlántico costero de Paraná, Brasil. In: Medellín RA, Equihua C, Chetkiewicz CLB, Crawshaw PGJ, Rabinowitz A, Redford KH, et al., editors. El jaguar en el nuevo milenio. Mexico: Fondo de Cultura Económica; 2002. p. 237-50.

16. Miranda EBP, Jacomo ATD, Torres NM, Alves GB, Silveira L. What are jaguars eating in a half-empty forest? Insights from diet in an overhunted Caatinga reserve. Journal of Mammalogy. 2018;99(3):724-31. doi: 10.1093/jmammal/gyy027. PubMed PMID: WOS:000434058500013.

17. Perilli MLL, Lima F, Rodrigues FHG, Cavalcanti SMC. Can Scat Analysis Describe the Feeding Habits of Big Cats? A Case Study with Jaguars (Panthera onca) in Southern Pantanal, Brazil. Plos One. 2016;11(3). doi: 10.1371/journal.pone.0151814. PubMed PMID: WOS:000372697400048.

18. Ramalho EE. Uso do habitat e dieta da onça-pintada (Panthera onca) em uma área de várzea, Reserva de Desenvolvimento Sustentável Mamirauá, Amazônia Central, Brasil. Brazil: Universidade Federal do Amazonas; 2006.

19. Silveira L. Ecologia comparada e conservação da onça-pintada (Panthera onca) e onça-parda (Puma concolor), no Cerrado e Pantanal. Brasília: Universidade de Brasília; 2004.

20. Sollmann R, Betsch J, Furtado MM, Hofer H, Jacomo ATA, Palomares F, et al. Note on the diet of the jaguar in central Brazil. European Journal of Wildlife Research. 2013;59(3):445-8. doi: 10.1007/s10344-013-0708-9. PubMed PMID: WOS:000321866400014.

21. Tirelli FP, de Freitas TRO, Michalski F, Percequillo AR, Eizirik E. Using reliable predator identification to investigate feeding habits of Neotropical carnivores (Mammalia, Carnivora) in a deforestation frontier of the Brazilian Amazon. Mammalia. 2019;83(5):415-27. doi: 10.1515/mammalia-2018-0106. PubMed PMID: WOS:000480513300001.

22. Trovati RG, Campos C, Brito B. Nota sobre convergência e divergência alimentar de canídeos e felídeos (Mamalia: Carnivora) simpátricos no Cerrado brasileiro. Neotropical Biology and Conservation. 2008;3:95-100.

23. Arroyo-Arce S, Thomson I, Cutler K, Wilmott S. Feeding habits of the jaguar Panthera onca (Carnivora: Felidae) in Tortuguero National Park, Costa Rica. Revista De Biologia Tropical. 2018;66(1):70-7. PubMed PMID: WOS:000425323900006.

24. Carrillo E, Fuller TK, Saenz JC. Jaguar (Panthera onca) hunting activity: effects of prey distribution and availability. J Trop Ecol. 2009;25:563-7. doi: 10.1017/s0266467409990137. PubMed PMID: WOS:000269168700014.

25. Chinchilla FA. Diets of Panthera onca, Felis concolor and Felis pardalis (Cartivora : Felidae) in Parque Nacional Corcovado, Costa Rica. Revista De Biologia Tropical. 1997;45(3):1223-9. PubMed PMID: WOS:000072230800031.

26. Gonzalez-Maya JF, Navarro-Arquez E, Schipper J. Ocelots as prey items of jaguars: a case from Talamanca, Costa Rica. Cat News. 2010;53:11-2.

27. Guadamuz VHM. Cambios en la abundancia, actividad temporal y dieta de jaguar (Panthera onca), otros felinos y sus presas en el Parque Nacional Santa Rosa, Área de Conservación Guanacaste, Costa Rica. Heredia, Costa Rica: Universidad Nacional de Costa Rica; 2012.

28. Estrada Hernández CG. Dieta, uso de hábitat y patrones de actividad del puma (Puma concolor) y el jaguar (Panthera onca) en la Selva Maya, Centroamerica. Revista Mexicana de Mastozoología. 2008;12:113-30.

29. Novack AJ, Main MB, Sunquist ME, Labisky RF. Foraging ecology of jaguar (Panthera onca) and puma (Puma concolor) in hunted and non-hunted sites within the Maya Biosphere Reserve, Guatemala. Journal of Zoology. 2005;267:167-78. doi: 10.1017/s0952836905007338. PubMed PMID: WOS:000233130900006.

30. Aranda M, SanchezCordero V. Prey spectra of jaguar (Panthera onca) and puma (Puma concolor) in tropical forests of Mexico. Studies on Neotropical Fauna and Environment. 1996;31(2):65-7. doi: 10.1076/snfe.31.2.65.13334. PubMed PMID: WOS:A1996WP94000001.

31. Avila-Najera DM, Palomares F, Chavez C, Tigar B, Mendoza GD. Jaguar (Panthera onca) and puma (Puma concolor) diets in Quintana Roo, Mexico. Animal Biodiversity and Conservation. 2018;41(2):257-66. doi: 10.32800/abc.2018.41.0257. PubMed PMID: WOS:000424865600006.

32. Cassaigne I, Medellin RA, Thompson RW, Culver M, Ochoa A, Vargas K, et al. Diet of pumas (Puma concolor) in Sonora, Mexico, as determined by GPS kill sites and molecular identified scat, with comments on jaguar (Panthera onca) diet. Southwestern Naturalist. 2016;61(2):125-32. PubMed PMID: WOS:000382172100005.

33. Cruz E. Current status of the jaguar in Chiapas. In: Ceballos G, Chávez C, List R, Zarza F, Medellín RA, editors. Jaguar Conservation and Management in Mexico: Case Studies and Perspectives. Mexico: Alianza WWF/Telcel-Universidad Nacional Autónoma de México; 2011. p. 81-9.

34. Gomez-Ortiz Y, Monroy-Vilchis O. Feeding ecology of puma Puma concolor in Mexican montane forests with comments about jaguar Panthera onca. Wildlife Biol. 2013;19(2):179-87. doi: 10.2981/12-092. PubMed PMID: WOS:000321679000008.

35. Gomez-Ortiz Y, Monroy-Vilchis O, Mendoza-Martinez GD. Feeding interactions in an assemblage of terrestrial carnivores in central Mexico. Zoological Studies. 2015;54. doi: 10.1186/s40555-014-0102-7. PubMed PMID: WOS:000351906000016.

36. Hernandez-SaintMartin AD, Rosas-Rosas OC, Palacio-Nunez J, Clemente-Sanchez F, Hoogesteijn AL. Food Habits of Jaguar and Puma in a Protected Area and Adjacent Fragmented Landscape of Northeastern Mexico. Natural Areas Journal. 2015;35(2):308-17. doi: 10.3375/043.035.0213. PubMed PMID: WOS:000353214800012.

37. Nunez R, Miller B, Lindzey F. Food habits of jaguars and pumas in Jalisco, Mexico. Journal of Zoology. 2000;252:373-9. doi: 10.1017/s095283690000011x. PubMed PMID: WOS:000165318600011.

38. Rosas-Rosas OC, Bender LC, Valdez R. Jaguar and puma predation on cattle calves in northeastern Sonora, Mexico. Rangeland Ecology & Management. 2008;61(5):554-60. doi: 10.2111/08-038.1. PubMed PMID: WOS:000259409400010.

39. Rueda P, Mendoza GD, Martinez D, Rosas-Rosas OC. Determination of the jaguar (Panthera onca) and puma (Puma concolor) diet in a tropical forest in San Luis Potosi, Mexico. Journal of Applied Animal Research. 2013;41(4):484-9. doi: 10.1080/09712119.2013.787362. PubMed PMID: WOS:000327469200017.

40. McBride RT, Giordano AJ, Ballard WB. Notes of the winter diet of jaguars (Panthera onca) in the transitional Paraguayan Chaco. Bellbird: Journal of Paraguayan Zoology. 2010;Bellbird 4 Accessed online.

41. Taber AB, Novaro AJ, Neris N, Colman FH. The food habits of sympatric jaguar and puma in the Paraguayan Chaco. Biotropica. 1997;29(2):204-13. doi: 10.1111/j.1744-7429.1997.tb00025.x. PubMed PMID: WOS:A1997XG77800009.

42. Emmons LH. Comparative feeding ecology of felids in a neotropical rainforest. Behavioural Ecology and Sociobiology. 1987;20(4):271-83.

43. Kuroiwa A, Ascorra C. Dieta y densida de posibles presas de jaguar en las inmediaciones de la zona de reserva Tambopata-Candamo, Perú. In: Medellín RA, Equihua C, Chetkiewicz CLB, Crawshaw PGJ, Rabinowitz A, Redford KH, et al., editors. El jaguar en el nuevo milenio. Mexico: Fondo de Cultura Económica; 2002. p. 199-208.

44. Farrell LE, Romant J, Sunquist ME. Dietary separation of sympatric carnivores identified by molecular analysis of scats. Molecular Ecology. 2000;9(10):1583-90. doi: 10.1046/j.1365-294x.2000.01037.x. PubMed PMID: WOS:000089998600013.

45. Scognamillo D, Maxit IE, Sunquist M, Polisar J. Coexistence of jaguar (Panthera onca) and puma (Puma concolor) in a mosaic landscape in the Venezuelan llanos. Journal of Zoology. 2003;259:269-79. doi: 10.1017/s0952836902003230. PubMed PMID: WOS:000182607100006.
