# Supplementary material for: Dietary similarity among jaguars (Panthera onca) in a high-density population
Source: PLoS One. 2022 Oct 10;17(10):e0274891. doi: 10.1371/journal.pone.0274891 (PMC9550027; doi:10.1371/journal.pone.0274891)
Supplement: Attachment — Submitted filename: Response-to-Reviewers-2022-05-17-RFoster.docx [file pone.0274891.s004.docx]

Rebecca Foster, PhD

Conservation Science, Panthera

Email. rfoster@panthera.org

Ph. (0044) (0)7941648239

17-May-2022

Dear Reviewer

We thank you for the constructive comments on our manuscript.

Please see our responses below:

Major fixes (line numbers refer to the marked up/tracked changes version)

(1) We have changed the title to ‘Diet similarity among jaguars (*Panthera onca*) in a high-density population’. We have deleted the sentence that implies that we will be investigating sex-based differences (lines 149). We have added the following ‘As long-term camera trap data from the study site revealed male-biased use of the trail system (Harmsen et al 2017), we anticipated a male bias in the scat data also.’ (lines 156-157).

(2) To clarify that ‘prey items’ refers to individuals not species, we have added ‘individual prey animals’ in parentheses after our first reference to ‘prey items’ in the Abstract (lines 21-22).

(3) We have removed the average prey item per scat for males and females, and replaced it with the average number of prey items per scat for jaguars; and we have included the standard deviation and range (lines 242-243).

(4) The Reviewer agrees that these results are suggestive and does not request we make any specific changes to the text; indeed, we already indicate that the results in this section are suggestive. To further clarify, we have rewritten the final sentence to make it clearer that while sample size bias explains much of the variation between individuals in the consumption of small prey and armadillos, it explains only a low amount of variation between individuals in the consumption rate of ungulates, suggesting that biological/ecological factors may be more relevant here (lines 347-351). Additionally, we have explicitly stated the sample sizes at which prey taxa reached an asymptote for each of the three scales of prey categorization (Figure 2, lines 288-298).

(5) We have shortened the paragraph about sex-based diet variations (lines 498-504).

(6) Evidence (camera-trap data) from the same study area shows that females only use the trail system sporadically and have lower detection probability than males, however there is no evidence of the population being skewed towards one sex, with long-term monitoring revealing a relatively even sex ratio (Harmsen et al 2017, Harmsen et al 2020). Therefore, as sampling for scats only occurred along trails, we consider that differential trail-use is a sufficient explanation for the male sampling-bias. We have rewritten the sentence to indicate that the sex ratio is not skewed (line 526).

(7) We have removed from the Conclusion that jaguar diet has changed in the study area. We have noted in the Discussion that armadillos have remained a core component of jaguar diet over a period of 20 years, citing Rabinowitz & Nottingham (1986), a study from the same study area, which has the third largest sample of jaguar scats to date in the literature (228 scats, Table S1), comparable in size to the number of scats used in this current study (322 scats), and considered large enough to reliably detect the principle prey taxa.

(8) We have shortened the Conclusion (deleted lines 643-701), and rewritten and moved arguments and references as supporting evidence to the Discussion (lines 406-453, 611-627).

(9) We have included some sampling recommendations in the Conclusion (lines 628-642).

Minor fixes

(1) We have corrected the scientific name of leopard

(2) We have deleted (*Panthera pardus*)

(3) Where referring to particular studies, we have mentioned the authors by name throughout
(4) We have deleted the repeated ‘this’
(5) We have changed ‘information one the’ to ‘information on the’

We believe that we have fully responded to all your comments. We look forward to hearing from you.

Yours faithfully

Rebecca Foster, PhD

**References**

Harmsen BJ, Foster RJ, Sanchez E, Gutierrez-Gonzalez CE, Silver SC, Ostro LET, et al. Long term monitoring of jaguars in the Cockscomb Basin Wildlife Sanctuary, Belize; Implications for camera trap studies of carnivores. Plos One. 2017;12(6). doi: 10.1371/journal.pone.0179505. PubMed PMID: WOS:000404607900028.

Harmsen BJ, Foster RJ, Quigley H. Spatially explicit capture recapture density estimates: Robustness, accuracy and precision in a long-term study of jaguars (Panthera onca). Plos One. 2020;15(6):19. doi: 10.1371/journal.pone.0227468. PubMed PMID: WOS:000540938100031.

Rabinowitz AR, Nottingham BG. Ecology and behaviour of the jaguar (Panthera onca) in Belize, Central America. Journal of Zoology. 1986;210:149-59.
