# Supplementary material for: Dietary similarity among jaguars (Panthera onca) in a high-density population
Source: PLoS One. 2022 Oct 10;17(10):e0274891. doi: 10.1371/journal.pone.0274891 (PMC9550027; doi:10.1371/journal.pone.0274891)
Supplement: Attachment — Submitted filename: Response-to-Editor-2022-08-03-RFoster.docx [file pone.0274891.s005.docx]

Rebecca Foster, PhD

Conservation Science, Panthera

Email. rfoster@panthera.org

Ph. (0044) (0)7941648239

03-August-2022

Dear Dr Cristescu

Thank you for taking over from the previous academic editor at such short notice and for the constructive comments on our manuscript entitled ‘Diet similarity among jaguars (*Panthera onca*) in a high-density population’.

We detail our responses to your comments below.

*L158: insert "is" after "this"* - Done

*L637: "sample" instead of "samples"* - Done

*L315-317: did you discuss that this result might be a reflection of smaller sample size for females than for males?* – We have edited the sentence to clarify this.

*Question: Did the scats confirmed as jaguar originate from adults, subadults, kittens also? The inclusion of scats from young jaguars in the analysis could provide partial explanation of the high taxonomic richness of prey items in the scat of male jaguars. This is something to consider adding to the Discussion* – This is an interesting point. Camera trap data from the same trail systems indicate that the trails are monopolised by adult males; this sex bias is reflected in the scats that we found. It seems likely that the scats are produced by the adult males that we detected on camera traps. We have no reason to believe that the high taxonomic richness in the scats is explained by the scats originating from young individuals.

*Fig. 1: why not standardize the data by dividing number of prey items by number of scats processed for each jaguar?* Thank you for the suggestion. The goal of Fig. 1 is to illustrate how a few individuals contributed greatly to the dataset, while many individuals contributed minimally to the dataset. As the unit of analysis is “prey item”, we feel that it is more useful to display the number of prey items contributed per individual, rather than the number of prey items standardised by the number of scats collected per individual.

*Fig. 2: add "scats" in brackets* - Because scats may contain differing numbers of prey items, the analysis was based on the number of prey items sampled, not the number of scats. Therefore, we have added “(prey items)” to the x-axis in Fig 2, rather than “(scats)”.

*Fig. 3: if you chose to include the jaguar ID for the data that fall outside the confidence interval bands, why not include it for all data that fall outside the bands? There are multiple data points in each figure panel that are not within the confidence interval bands. Or are these the individuals with large residuals (>2 standard residuals) as per L231-233?* - Correct, these are the individuals with large residuals (>2 standard residuals). We have included this in the legend for clarity.

*Supplementary material: update manuscript title to the new manuscript* - Done

We believe that we have fully responded to all the comments. We look forward to hearing from you.

Yours faithfully

Rebecca Foster, PhD
